# Calcium-binding protein TgpCaBP regulates calcium storage of the zoonotic parasite *Toxoplasma gondii*

**DOI:** 10.1128/spectrum.00661-24

**Published:** 2024-08-20

**Authors:** Weisong Sun, Ning Jiang, Qilong Li, Yize Liu, Yiwei Zhang, Ran Chen, Ying Feng, Xiaoyu Sang, Shaojun Long, Qijun Chen

**Affiliations:** 1 Key Laboratory of Livestock Infectious Diseases, Ministry of Education, and Key Laboratory of Ruminant Infectious Disease Prevention and Control (East), Ministry of Agriculture and Rural Affairs, College of Animal Science and Veterinary Medicine, Shenyang Agricultural University, Shenyang, China; 2 Research Unit for Pathogenic Mechanisms of Zoonotic Parasites, Chinese Academy of Medical Sciences, Shenyang, China; 3 National Key Laboratory of Veterinary Public Health Security and College of Veterinary Medicine, China Agricultural University, Beijing, China; Clemson University, Clemson, South Carolina, USA

**Keywords:** *Toxoplasma gondii*, TgpCaBP, Ca^2+ ^signaling, parasite biology, calcium binding

## Abstract

*Toxoplasma gondii*, the causative parasite of toxoplasmosis, is an apicomplexan parasite that infects warm-blooded mammals. The ability of the calcium-binding proteins (CBPs) to transport large amounts of Ca^2+^ appears to be critical for the biological activity of *T. gondii*. However, the functions of some members of the CBP family have not yet been deciphered. Here, we characterized a putative CBP of *T. gondii*, TgpCaBP (TGME49_229480), which is composed of four EF-hand motifs with Ca^2+^-binding capability. TgpCaBP was localized in the cytosol and ER of *T. gondii*, and parasites lacking the *TgpCaBP* gene exhibited diminished abilities in cell invasion, intracellular growth, egress, and motility. These phenomena were due to the abnormalities in intracellular Ca^2+^ efflux and ER Ca^2+^ storage, and the reduction in motility was associated with a decrease in the discharge of secretory proteins. Therefore, we propose that TgpCaBP is a Ca^2+^ transporter and signaling molecule involved in Ca^2+^ regulation and parasitization in the hosts.

**IMPORTANCE:**

Ca^2+^ signaling is essential in the development of *T. gondii*. In this study, we identified a calcium-binding protein in *T. gondii*, named TgpCaBP, which actively regulates intracellular Ca^2+^ levels in the parasite. Deletion of the gene coding for TgpCaBP caused serious deficits in the parasite’s ability to maintain a stable intracellular calcium environment, which also impaired the secretory protein discharged from the parasite, and its capacity of gliding motility, cell invasion, intracellular growth, and egress from host cells. In summary, we have identified a novel calcium-binding protein, TgpCaBP, in the zoonotic parasite *T. gondii*, which is a potential therapeutic target for toxoplasmosis.

## INTRODUCTION

*Toxoplasma gondii*, an obligate intracellular apicomplexan parasite that infects approximately 33% of the global population (1), causes severe diseases in immunocompromised and congenitally infected individuals (2, 3). Calcium has been recognized as a key secondary messenger that triggers cell signaling cascades that affect the motility and secretion of proteins required for parasite invasion (4–6). During initial attachment of *T. gondii* parasites to host cells, secretory organelles located at the anterior of the parasite release contents of micronemes (7–9), which is regulated by cytoplasmic free calcium. Thus, calcium ions are essential for *T. gondii* motility and host cell invasion.

The endoplasmic reticulum (ER) is the largest store of calcium in *T. gondii,* and the SERCA-type Ca^2+^-ATPase expressed in the ER is responsible for regulating the uptake of calcium ions (10, 11). Calcium ion levels in *T. gondii* are dynamic and relatively stable and are maintained by CBPs (12, 13). CBPs, which are either calcium-sensitive or calcium-buffering proteins, can regulate the release or uptake of calcium from intracellular calcium storage organelles or the extracellular environment (11).

The structures of CBPs have been extensively studied in both mammals and plants (14, 15). The binding of most CBPs to calcium is believed to be highly dependent on the conserved EF-hand domains within the molecule. EF-hand domains have been shown to bind Ca^2+^, and proteins with EF-hand domain mutations are resistant to Ca^2+^-induced conformational alterations (16, 17). Analysis of both *T. gondii* (ToxoDB) and *Plasmodium falciparum* (PlasmoDB) genomes revealed 74 potential EF-hand domain-containing CBPs in *T. gondii*, and 103 homologous proteins in *P. falciparum* (18). CBPs with EF-hand domains include the calmodulin (CaM) family, the calcium-dependent protein kinase (CDPK) family, and the calcineurin B-like (CBL) family. Of these proteins, *T. gondii* CaM is the only protein that has been cloned and shown to bind Ca^2+^ *in vitro* (19). Previous studies have shown that CaM inhibitors impaired host cell invasion and intracellular development of both *T. gondii* and *P. falciparum* (20, 21). In addition, the CDPK family is found only in plants and protists and has been implicated in parasite motility, secretion, invasion, egress, and division (22–24). However, no CBL family proteins have been identified in *T. gondii*. Moreover, CBPs also include heat shock proteins, centrin/troponin C-like proteins, and C2 domain-containing proteins (25, 26).

In this study, we discovered an EF-hand domain-containing CBP with calcium-binding and transporting activities, TgpCaBP, located in the cytosol and ER of *T. gondii*. After deletion of the *TgpCaBP* gene using the CRISPR/Cas9 technique, the *ΔTgpCaBP* parasites showed reduced cell invasion, motility, intracellular growth, microneme secretion and egress abilities.

## RESULTS

### TgpCaBP contains a typical calcium-binding structure and is localized in the cytosol and endoplasmic reticulum of *T. gondii*

**Fig 1:**
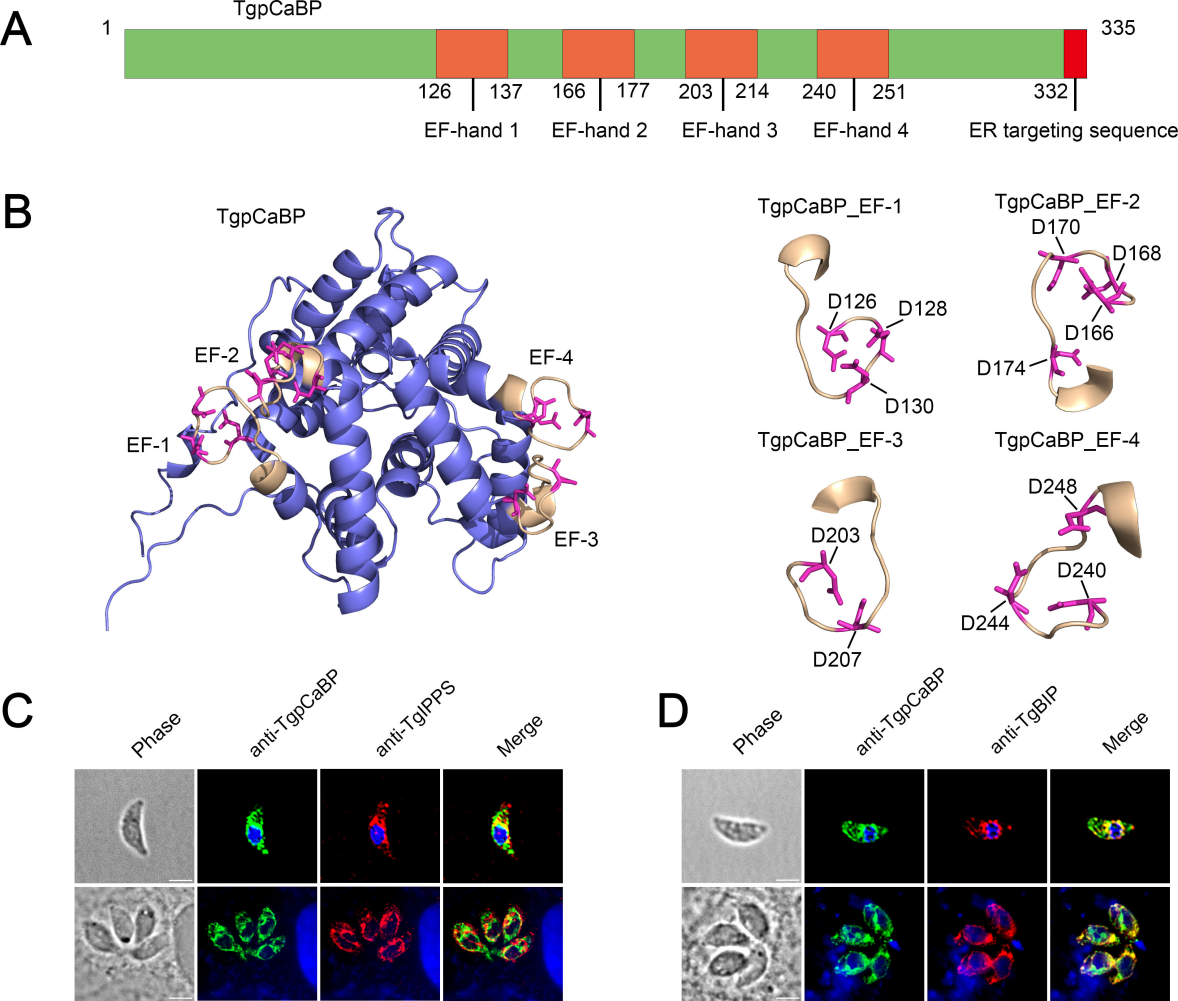
TgpCaBP contains a typical calcium-binding domain and is localized in the cytosol and endoplasmic reticulum of *T. gondii*. (**A**) A schematic presentation of the domain annotation of TgpCaBP of *T. gondii*. The predicted EF-hand domains are highlighted in orange color. An ER-targeting sequence located at the C-terminus is indicated in red color. (**B**) Structure modeling of TgpCaBP. Left: The structure of TgpCaBP is shown with EF-hand motifs, which consist of 12 amino acid residues (yellow) and conserved Asp residues (red). Right: Enlarged views of the EF-hand motifs of TgpCaBP showing the numbers of Asp residues. (**C, D**) Immunofluorescence assay (IFA) of TgpCaBP in the extracellular and intracellular tachyzoites using a rat anti-TgpCaBP antibody (1:100). TgpCaBP is localized in both cytosol and endoplasmic reticulum and co-localized with the anti-TgIPPS and TgBIP antibodies. Scale bar, 5 µm.

We screened the genes that potentially encode CBPs in the *T. gondii* database (ToxoDB). Of which, a gene (TGME49_229480) encodes a putative CBP (TgpCaBP) with 335 amino acids and a molecular weight of 36 kDa was identified. TgpCaBP comprises four EF-hand motifs and an ER-targeting sequence (Fig. 1A). We used ESPript 3.0 software to compare the amino acid sequences of TgpCaBP with other EF-hand domain-containing proteins. TgpCaBP was found to share similarities in the EF-hand domains and the C-terminal HDEL with two human ER-resident EF-hand calcium-binding proteins, reticulocalbin (27), ERC-55 (28), and a *Plasmodium falciparum* ER-resident EF-hand calcium-binding protein called PfERC (29). The consensus sequences for an EF-hand loop are composed of animal acids, including D, E, N, S, or T (Fig. S1A). Homology modeling showed that the conserved EF-hand motifs formed Asp-rich pockets that likely bind calcium (Fig. 1B). In addition, the transcriptional level and protein expression of TgpCaBP in RH strain were higher than that in the ME49 strain (Fig. S2A and B).

The presence of HDEL sequence in TgpCaBP indicates compartmentalization in the ER, and immunofluorescence experiments with a protein-specific antibody showed that TgpCaBP was localized in the cytosol and ER of the parasites (Fig. 1C and D). Localization of TgpCaBP in the ER was further confirmed by the co-localization of two ER protein markers, decaprenyl diphosphate synthase (IPPS), and binding immunoglobulin protein (BIP) (30), with protein-specific antibodies (Fig. 1C and D). These results revealed the localization of TgpCaBP in both the cytosol and ER of the parasite.

### TgpCaBP was highly soluble in the presence or absence of calcium

**Fig 2:**
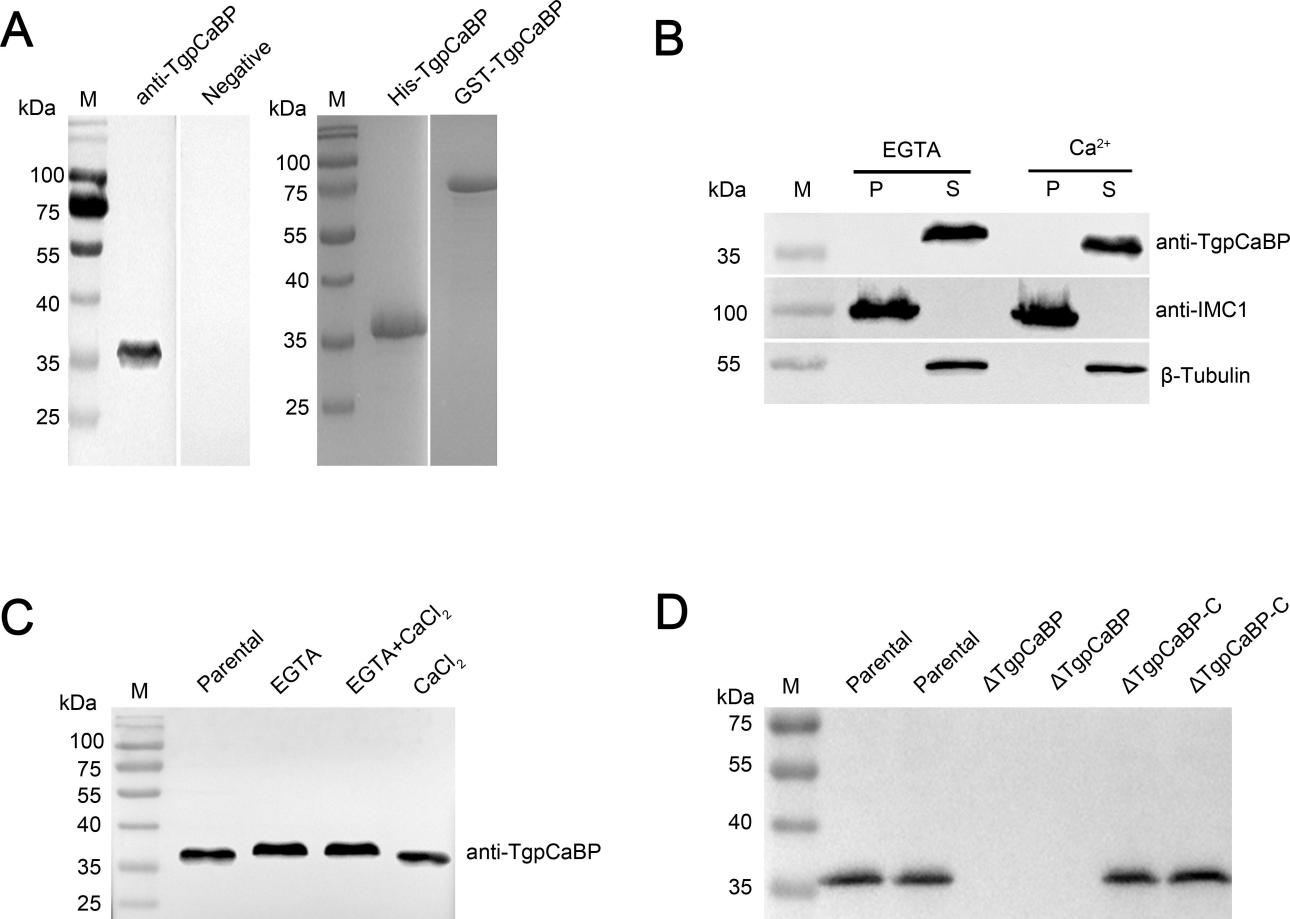
Calcium binding property of TgpCaBP. (**A**) Western blot analysis of native, HIS- and GST-tagged TgpCaBP using a rat anti-TgpCaBP antibody. (**B**) Calcium-dependent solubility was detected using western blotting. Parasites were lysed in 1% Triton X-100 in 5 mM EGTA or 5 mM CaCl_2_ and fractionated by centrifugation. TgpCaBP was detected with a rat anti-TgpCaBP antibody, a mouse anti-IMC1 antibody was used as a control for the protein in the pellet (P) and a rabbit β-Tubulin monoclonal antibody was used as a control for the protein in the supernatant (S). (**C**) TgpCaBP migrated faster in the presence of Ca^2+^. Ca^2+^ was replaced by Mg^2+^ and K^+^ in the control group, and EGTA was used to chelate-free calcium in the extracellular environment (Fig. S3A and B). Parasites were lysed in 1% Triton X-100 and incubated with EGTA, a combination of EGTA and CaCl_2_, or CaCl_2_. The native protein was extracted from *T. gondii* RH tachyzoites. (**D**) The expression of TgpCaBP in the parental, *ΔTgpCaBP,* and *ΔTgpCaBP-C* parasites (with two batches of proteins from each parasite line) was analyzed using western blotting with a rat anti-TgpCaBP antibody to verify the presence and absence of TgpCaBP protein in different parasite lines.

First, recombinant His-TgpCaBP (35.5 kDa) and GST-TgpCaBP (61.5 kDa) fusion proteins were generated by the prokaryotic expression system (Fig. 2A). The TgpCaBP-specific antibodies were generated by immunizing rats and rabbits with the His-tagged TgpCaBP fusion protein. These antibodies were then used to investigate the cellular localization of TgpCaBP. The intact native TgpCaBP protein with an approximate molecular weight of 36 kDa was detected using a specific rat anti-TgpCaBP antibody by western blotting (Fig. 2A). The specificity of the anti-TgpCaBP antibody was also verified with proteins from TgpCaBP-knockout *T. gondii*. To examine the calcium interaction with TgpCaBP, we analyzed the soluble (cytosolic) and insoluble (cytoskeleton) components of the parasite (16) and assessed the distribution of TgpCaBP in the presence of calcium or EGTA. TgpCaBP was soluble with or without calcium (Fig. 2B). Inner membrane complex protein 1 (IMC-1) was used to identify the insoluble cytoskeleton. In addition, the solubility test found that TgpCaBP migrated faster after binding Ca^2+^, and subsequent experiment confirmed this property (Fig. 2C). In addition, there was no significant change in the electrophoretic mobility of TgpCaBP between the parental group and the CaCl_2_ group, which may be because the calcium ions contained in the parasites can saturate the protein-bound calcium ions and cause conformational changes (Fig. 2C). Furthermore, EGTA was used to chelate free calcium in the extracellular environment to eliminate the influence of calcium on electrophoretic mobility, and it was found that the electrophoretic mobility of TgpCaBP was not affected by Mg^2+^ or K^+^ (Fig. S3A and B).

### TgpCaBP is essential for gliding, host cell invasion, intracellular growth, and egress of *T. gondii*

To investigate the biological role of TgpCaBP in *T. gondii*, we generated a *TgpCaBP* gene-deleted parasite line (*ΔTgpCaBP*) using the CRISPR-Cas9 gene editing technique, in which the *TgpCaBP* genomic locus was replaced with a gene cassette for the dihydrofolate reductase-thymidylate synthase (DHFR) (Fig. S4A). The precise replacement of *TgpCaBP* with *DHFR* was confirmed using PCR (Fig. S4B) and quantitative real-time PCR (Fig. S4E). Furthermore, we generated a complementary cell line *ΔTgpCaBP-C* with a pSAG1-CAS9-U6-sgTgpCaBP plasmid that contains the coding sequence (CDS) of the *TgpCaBP* gene (Fig. S4C). Verification of complete *TgpCaBP* deletion in the gene knockout strain and precise gene insertion in the complementary strain was conducted using PCR (Fig. S4D) and western blotting, which further confirmed that the *TgpCaBP* gene was absent in the *ΔTgpCaBP* parasite and present in the *ΔTgpCaBP-C* (Fig. 2D), quantitative PCR (qPCR) and IFA were used to further determine the absent expression of *TgpCaBP* and its complementation in the respective parasite lines (Fig. S4E and F).

**Fig 3:**
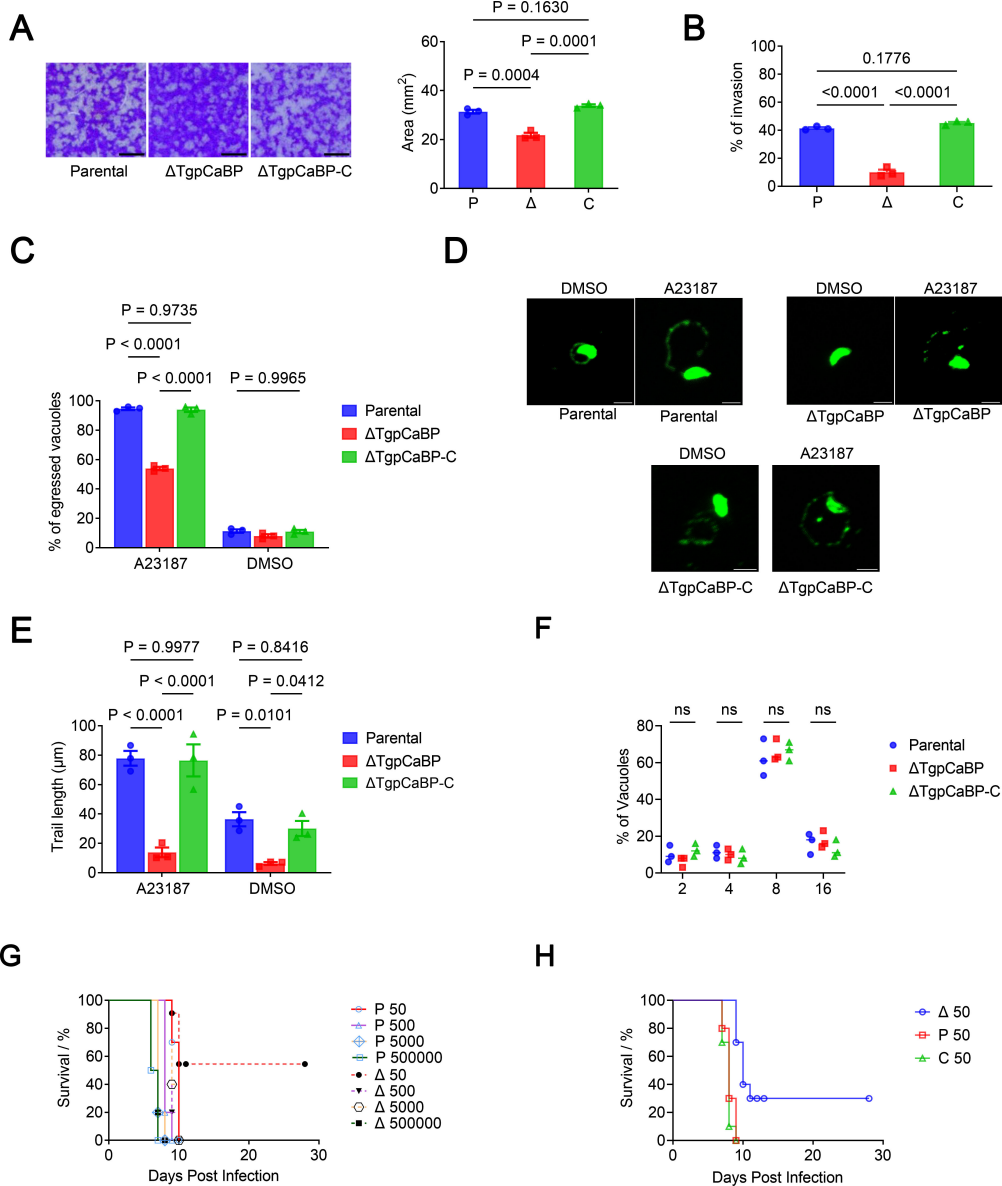
TgpCaBP was associated with intracellular growth and gliding motility of *T. gondii*. (**A**) Plaque assays were conducted for parental (P), *ΔTgpCaBP* (*Δ*), and *ΔTgpCaBP-C* (C) parasites. Quantification of plaque sizes from three independent biological experiments using one-way ANOVA with Tukey’s multiple comparisons. Values are means ± SEM; (*n* = 3). *P* > 0.05: not significant. Scale bar, 2 mm. (**B**) Red/green assays of parental, *ΔTgpCaBP,* and *ΔTgpCaBP-C* parasites calculating the invasion efficiency. The invasion capacity of the *ΔTgpCaBP* parasites was significantly lower than that of the parental and the gene-complemented parasites. Quantification of invasion from three independent biological experiments using one-way ANOVA with Tukey’s multiple comparisons. Values are means ± SEM; (*n* = 3). *P* > 0.05: not significant. (**C**) Egress assays of the parental, *ΔTgpCaBP,* and *ΔTgpCaBP-C* parasites stimulated by 3 µM A23187. The egress of the *ΔTgpCaBP* parasites was significantly lower than the other two parasite lines. Quantification of egress from three independent biological experiments using two-way ANOVA with Tukey’s multiple comparisons. Values are means ± SEM; (*n* = 3). *P* > 0.05: not significant. (**D**) Effect of A23187 on gliding of *T. gondii*. Indirect immunofluorescence microscopy demonstrated that the length of trails deposited during gliding with A23187 treatment in the parental and *ΔTgpCaBP-C* parasite was longer and more complete than the *ΔTgpCaBP* parasite. Trails were visualized with a mouse anti-SAG1 antibody and conjugated to Alexa 488. Scale bar, 5 µm. (**E**) Quantification and statistical analysis of the trail length of the parental, *ΔTgpCaBP,* and *ΔTgpCaBP-C* parasites treated with A23187 and dimethyl sulfoxide control. The motility was impaired in the *ΔTgpCaBP* parasites and rescued in the *ΔTgpCaBP-C* parasites. Values are means ± SEM; (*n* = 3). *P* > 0.05: not significant. (**F**) Replication assays of the parental, *ΔTgpCaBP,* and *ΔTgpCaBP-C* parasites. There was no significant difference between the replication of the *ΔTgpCaBP* parasites and the other two parasite lines. Quantification of replication from three independent biological experiments using two-way ANOVA with Tukey’s multiple comparisons. Values are means ± SEM; (*n* = 3). Ns: not significant. (**G**) Survival of mice challenged with 50, 500, 5 × 10^3^, or 5 × 10^5^ parental and *ΔTgpCaBP* parasites. (**H**) Survival of mice challenged with 50 parental, *ΔTgpCaBP,* and *ΔTgpCaBP-C* parasites. The virulence of the *ΔTgpCaBP* parasites decreased significantly compared to the parental parasites. The virulence was rescued in the *ΔTgpCaBP-C* parasites. All mice were monitored for 28 days. All data were analyzed using simple survival analysis (Kaplan-Meier), *P* < 0.0001.

Next, the function of *TgpCaBP* associated with *T. gondii* growth was evaluated using plaque assays, in which the parasites undergo repeated cycles of invasion, replication, and egress, causing host cell lysis. The *ΔTgpCaBP* parasites proliferated much slower represented by the formation of smaller plaques compared to those formed by their parental counterparts (Fig. 3A). Notably, the genetically complemented parasites, *ΔTgpCaBP-C*, partially rescued from this defect (Fig. 3A).

To determine the involvement of TgpCaBP in the cell lytic cycle of *T. gondii*, we compared the efficiency of invasion, egress, and replication among the wild type (WT) of RH strain (Parental), the *ΔTgpCaBP,* and the *ΔTgpCaBP-C* parasites. A red/green invasion assay for the invasion efficiency test to discriminate between extracellular and intracellular parasites was used as previously described (31). The *ΔTgpCaBP* parasites showed a lower invasion capability than that of the parental and gene-complemented parasite lines (Fig. 3B). To analyze egress, a calcium ionophore, A23187, was used to increase cytosolic calcium levels of the parasites (32), and the concentration of A23187 was set to 3 µM. The results revealed that the *ΔTgpCaBP* parasites showed a longer duration before egress from the host cells than that of the parental and the *ΔTgpCaBP-C* lines (Fig. 3C). Furthermore, the parental, *ΔTgpCaBP* and *ΔTgpCaBP-C* parasites were stimulated with 3 µM A23187 to observe its effect on parasite gliding ability; the result indicated that the mobility of the *ΔTgpCaBP* parasites were reduced, and the mobility phenotype was rescued in the *ΔTgpCaBP-C* parasites. In addition, the average trail length of *ΔTgpCaBP* parasites was significantly reduced than that of the parental parasites (Fig. 3D and E). The striking differences in the gliding motility of the parental, the *ΔTgpCaBP* parasites, and the *ΔTgpCaBP-C* parasites were also observed with time-lapse microscopy (Supporting Videos S1 to S3).

Even though there were no significant differences in *in vitro* replication among the parental, the *ΔTgpCaBP* and the *ΔTgpCaBP-C* parasites (Fig. 3F), the virulence of the *ΔTgpCaBP* parasites to mice was significantly reduced (Fig. 3G), and importantly, the *ΔTgpCaBP-C* parasites recovered their virulence (Fig. 3H).

### Deletion of *TgpCaBP* impaired microneme secretion

**Fig 4:**
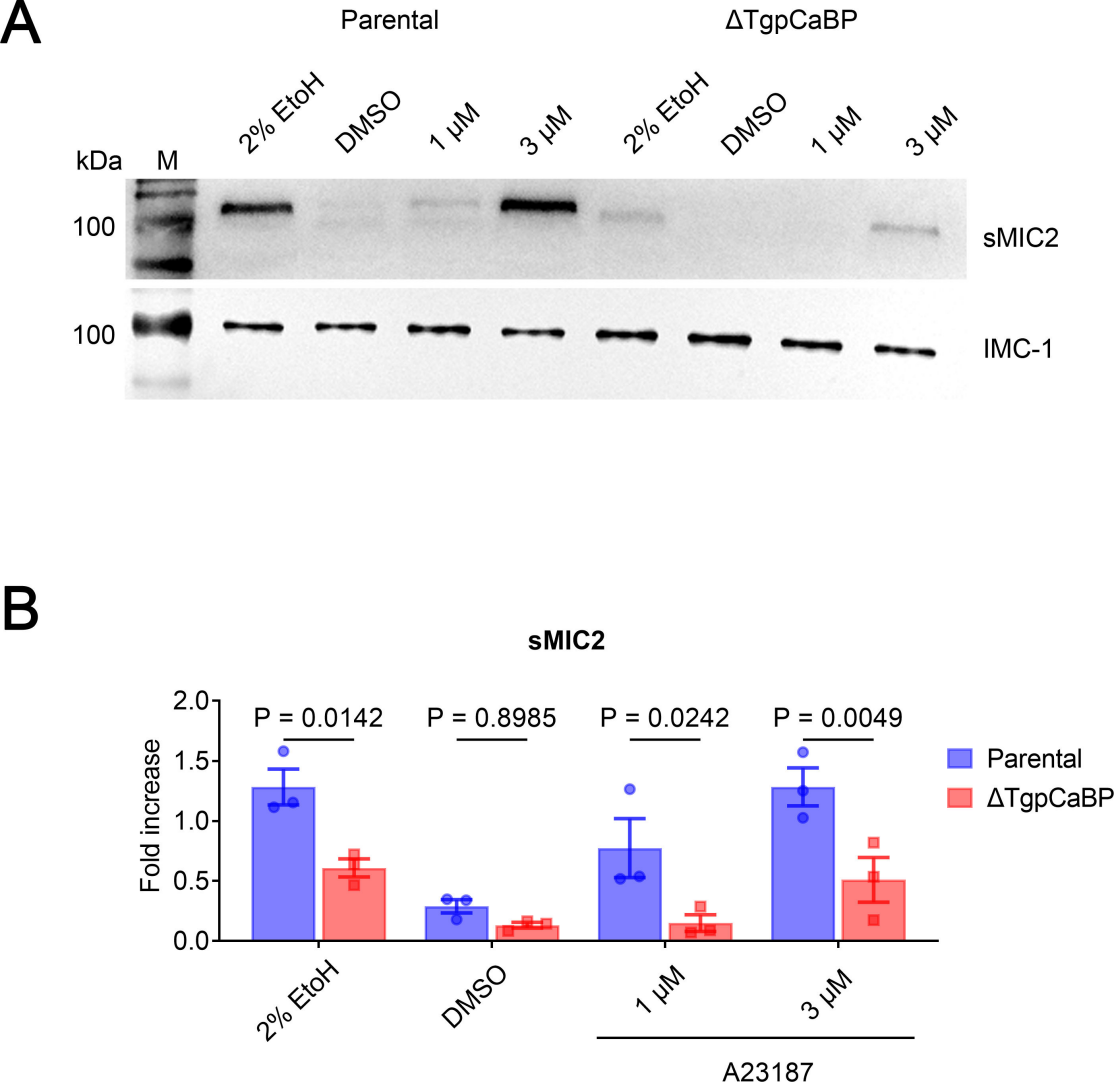
Effect of the Ca^2+^ ionophore A23187 on microneme secretion of *T. gondii*. (**A**) Western blot of supernatants secreted by parasites after stimulation with A23187 at 37°C for 5 min, showing a significant difference in MIC2 secreted by the parental and the *ΔTgpCaBP* parasites. The pellets were monitored by a mouse anti-IMC-1 monoclonal antibody. Stimulation with 2% ethanol was used as a positive control, and DMSO was used as a solvent control. (**B**) Quantification and statistical analysis of MIC2 secreted by the parental and the *ΔTgpCaBP* parasites after treatment with 2% ethanol, DMSO, and different concentrations of A23187. Secretion of MIC2 was inhibited in the *ΔTgpCaBP* parasites. Values are means ± SEM; (*n* = 3). *P* > 0.05: not significant.

To determine the involvement of TgpCaBP in microneme secretion of the parasite, tachyzoites of the parental and the *ΔTgpCaBP* parasites were briefly treated with various concentrations of Ca^2+^ ionophore A23187, which potently increases cytosolic Ca^2+^ concentration, with 2% ethanol as a positive control and dimethyl sulfoxide (DMSO) as a solvent control. MIC2 secreted from the parasites was examined using western blotting. MIC2 has been used as a marker for microneme secretion; secreted MIC2 (sMIC2) is released into the supernatant. The pellets were monitored based on IMC-1. Results showed that the secretion of microneme protein 2 (sMIC2) in the parental parasites was induced by the treatment with A23187. Compared to the parental parasites, the *ΔTgpCaBP* parasites showed a reduced secretion of invasion-associated proteins, especially with the treatment of 1 and 3 µM A23187, and 2% ethanol (Fig. 4A). Quantitative analysis of MIC2 secretion of the parental and *ΔTgpCaBP* parasites showed that the MIC2 secretion between the parental and *ΔTgpCaBP* parasites was significantly different under stimulation with 2% ethanol, 1 and 3 µM A23187 (Fig. 4B).

### TgpCaBP is an important regulatory protein of cytosolic Ca^2+^

**Fig 5:**
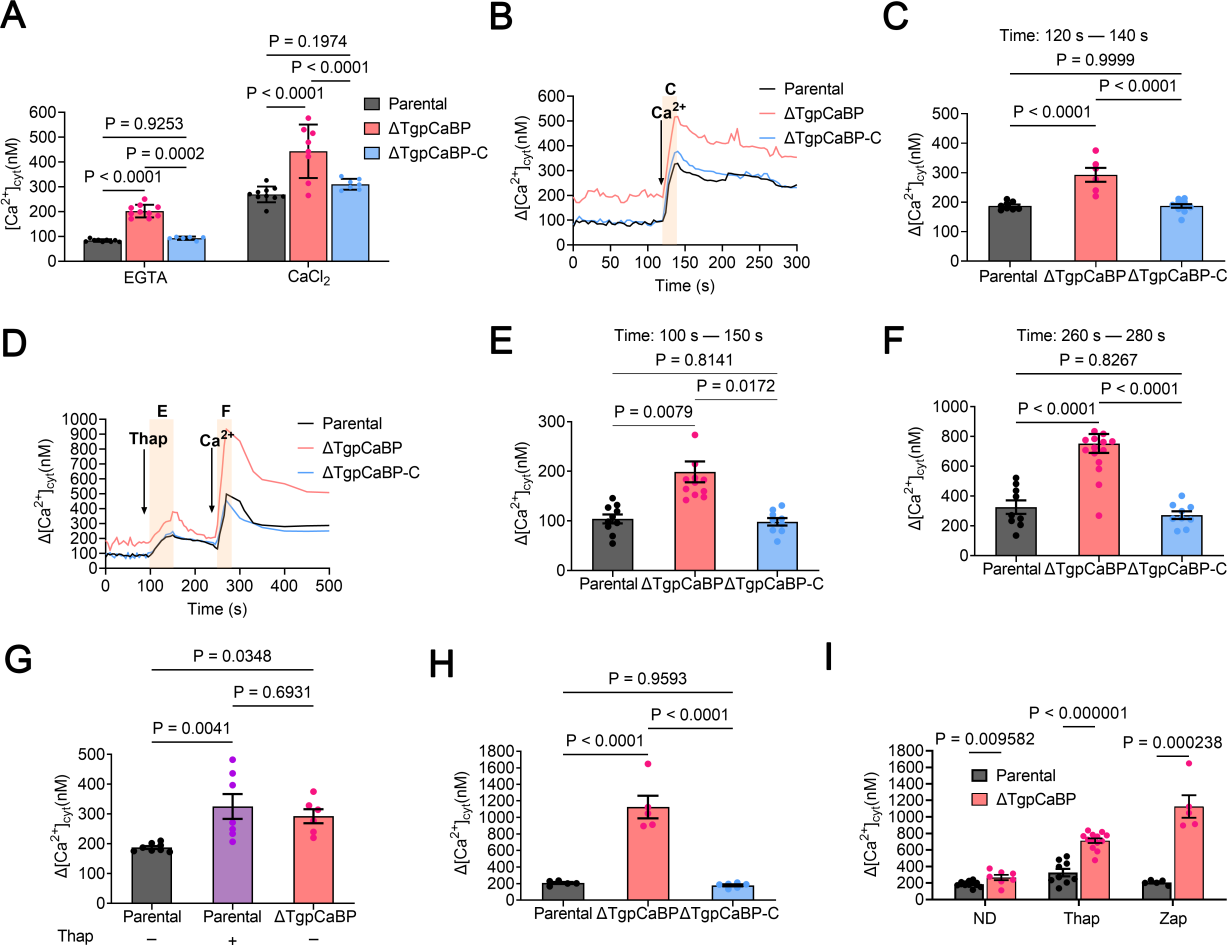
Role of TgpCaBP in the efflux of cytosolic Ca^2+^ of *T. gondii*. (**A**) Intracellular calcium concentration ([Ca^2+^]_i_) (nM) of the parental parasites, the *ΔTgpCaBP* and the *ΔTgpCaBP-C* parasites in the presence of 100 µM EGTA or 2 mM CaCl_2_. Values are means ± SEM; (*n* = 6–10). *P* > 0.05: not significant. (**B**) Measurements of dynamic cytosolic Ca^2+^ in the presence of Fura-2AM in tachyzoites of the parental (RH), the *ΔTgpCaBP* and the *ΔTgpCaBP-C* parasites. The buffer contains 100 µM EGTA to chelate contaminating Ca^2+^, and 2 mM Ca^2+^ was added to the suspension at 120 s. The pink box indicates the area used for the quantification presented in (**C**). (**C**) Quantification and statistical analysis of the change in cytosolic Ca^2+^ during the first 20 s after the addition of extracellular Ca^2+^. Values are means ± SEM; (*n* = 7–12). *P* > 0.05: not significant. (**D**) Cytosolic Ca^2+^ increases after adding Thap (1 µM), following Ca^2+^ influx resulting from adding 2 mM extracellular Ca^2+^ at 260 s. Thap: thapsigargin. The pink boxes indicate the area used for the quantification presented in (**E**) and (**F**). (**E**) Quantification and statistical analysis of the change in cytosolic Ca^2+^(Δ[Ca^2+^]_cyt_) at 50 s after adding Thap. Values are means ± SEM; (*n* = 9–12). *P* > 0.05: not significant. (**F**) Quantification and statistical analysis of the Δ[Ca^2+^]_cyt_ at 20 s after adding 2 mM of Ca^2+^. Values are mean ± SEM; (*n* = 9–16). *P* > 0.05: not significant. (**G**) Quantification and statistical analysis of the Δ[Ca^2+^]_cyt_ at 20 s after adding Ca^2+^ in the presence or absence of Thap in the parental (P) and the *ΔTgpCaBP* parasites. Values are means ± SEM; (*n* = 7–12). *P* > 0.05: not significant. (**H**) Quantification and statistical analysis of cytosolic Ca^2+^ increase stimulated by Zap (100 µM) in the presence of 2 mM extracellular Ca^2+^. Values are means ± SEM; (*n* = 5–6). *P* > 0.05: not significant. Zap: zaprinast. (**I**) Quantification and statistical analysis of the Δ[Ca^2+^]_cyt_ during the 20 s after adding Ca^2+^ without additions (ND) or after adding Thap or Zap. Values are means ± SEM; (*n* = 5–15). *P* > 0.05: not significant. Two-way ANOVA with Tukey’s multiple comparison test for A and I and one-way ANOVA with Tukey’s multiple comparison tests for C, E, F, G, and H.

To determine whether deletion of *TgpCaBP* would influence the maintenance of intracellular Ca^2+^ balance of the parasite, we measured basal cytosolic Ca^2+^ concentration in extracellular tachyzoites pretreated with Fura-2 AM in the presence of 100 µM EGTA. The result showed that the concentration of cytosolic Ca^2+^ in the *ΔTgpCaBP* parasites was the highest of all parasite lines (Fig. 5A). Next, the cytosolic Ca^2+^ changes after adding 2 mM Ca^2+^ were evaluated. After subjecting all three parasite lines to a 2 mM Ca^2+^ pulse, cytosolic Ca^2+^ levels increased, with the most significant increase observed in the *ΔTgpCaBP* parasites (Fig. 5B and C). TgpCaBP probably exhibits a Ca^2+^ regulatory function by effluxing excess Ca^2+^ from the parasite; however, this function was impaired in the *ΔTgpCaBP* parasite.

To investigate whether the Ca^2+^-activated-Ca^2+^ entry (CACE) mechanism (33) was affected, we added thapsigargin (TG) to tachyzoites in suspension, which inhibits the SERCA-type Ca^2+^-ATPase of the parasites and causes Ca^2+^ to leak from the ER due to the blocked uptake pathway. Treatment of tachyzoites with TG resulted in an increase in Ca^2+^ in the cytoplasm due to the inhibition of the SERCA Ca^2+^-ATPase in the ER, which led to uncompensated Ca^2+^ efflux into the cytosol. Furthermore, the increased intracellular Ca^2+^ concentration further promoted extracellular Ca^2+^ influx at the PM in the parental and the *ΔTgpCaBP* parasites, indicating normal CACE activity (Fig. 5D). The rate of Ca^2+^ rising (Δ[Ca^2+^]_cyt_) was measured as the change in Ca^2+^ concentration after adding TG (Fig. 5E) or Ca^2+^ (Fig. 5F). Notably, the Δ[Ca^2+^]_cyt_ of the parental parasites shown in Fig. 5F (black column) was approximately 1.7 times as high as that measured with no added TG (Fig. 5C). However, the Δ[Ca^2+^]_cyt_ of the *ΔTgpCaBP* parasite (Fig. 5F) was approximately 2.8 times as high as that measured without the addition of TG (Fig. 5C). The significant elevation of intracellular Ca^2+^ in the *ΔTgpCaBP* parasites suggests that the deletion of *TgpCaBP* impaired the regulatory mechanism of intracellular Ca^2+^ of the parasites. When extracellular Ca^2+^ was added to both the parental and *ΔTgpCaBP* parasites, the Δ[Ca^2+^]_cyt_ in the TG-treated parental was similar to that in the untreated *ΔTgpCaBP* parasites (Fig. 5C, F, and G). These results implied that the *ΔTgpCaBP* parasites lost the function of transporting Ca^2+^ into the ER, leading to impaired ER calcium storage; however, this function was restored in the *ΔTgpCaBP-C* parasites.

In addition, the effects of the phosphodiesterase inhibitor zaprinast (Zap), which increases cytosolic Ca^2+^ by modulating cyclic nucleotide cGMP increase (34, 35), were tested. The addition of Zap resulted in a higher Δ[Ca^2+^]_cyt_ increase in the *ΔTgpCaBP* parasites (Fig. 5H) than that with or without TG treatment (ND) (Fig. 5I). This further suggested that the *ΔTgpCaBP* parasites lost the regulatory ability of Ca^2+^ efflux.

## DISCUSSION

Many studies have revealed that intracellular Ca^2+^ modulates various cellular processes including cell proliferation and protein secretion in both eukaryotes and prokaryotes (11, 26, 36), and CBPs, located in the cellular lumens, play central roles in these processes. CBPs are heterogeneous proteins that bind Ca^2+^ rapidly and reversibly through their highly conserved EF-hand domains, which consist of a Ca^2+^-coordinating loop flanked by two alpha-helices (11). The most prevalent EF-hand domains of CBP can be divided into canonical and pseudo-domains (12). CBPs containing canonical domains consisting of pairs of EF-hand domains are associated with Ca^2+^ transduction, serving as Ca^2+^ buffering proteins. Conversely, CBPs containing pseudo-domains consisting of an uneven number of EF-hand domains play a role in Ca^2+^ transduction as Ca^2+^ sensing proteins (12, 37). Notably, calcium-buffering proteins have a higher affinity for Ca^2+^ than calcium-sensing proteins. Furthermore, calcium-sensing proteins are generally Ca^2+^ channel proteins that undergo a conformational change upon binding to calcium, resulting in a significant opening of their structure, allowing calcium entry and interaction with downstream targets (17). Therefore, they determine the entry of Ca^2+^ and regulate the time needed to reach a steady state without affecting the final level. However, when calcium buffering proteins maintain a “relative closed” conformation when they bind to Ca^2+^, consequently achieving significant Ca^2+^ distribution into the cytosol or storage organelles such as the ER, the largest store of Ca^2+^ in cells (36).

This study identified and functionally analyzed a novel CBP in *T. gondii*, namely TgpCaBP, which has four EF-hand motifs and an ER-targeting sequence (HDEL) (Fig. 1). The EF-hand domains and C-terminal HDEL sequence of TgpCaBP are similar to other EF-hand calcium-binding proteins. The study revealed that TgpCaBP is highly soluble and Ca^2+^ can affect its electrophoretic mobility (Fig. 2); however, Mg^2+^ and K^+^ did not exhibit this feature (Fig. S3A). Immunofluorescence results showed that TgpCaBP was localized in the cytosol and ER of intracellular and extracellular tachyzoites (Fig. 1). These data implied that TgpCaBP may regulate intracellular Ca^2+^ signaling.

The cytosolic Ca^2+^ in parental, *ΔTgpCaBP,* and *ΔTgpCaBP-C* parasites treated with Ca^2+^ indicated elevated Ca^2+^ levels in the cytoplasm. Notably, the level of Ca^2+^ in the *ΔTgpCaBP* parasite remained significantly higher despite the decline in the intracellular Ca^2+^ levels (Fig. 5A through D), indicating that TgpCaBP plays a critical role in Ca^2+^ regulation and homeostasis. Previous studies indicated that the sarcoendoplasmic Ca^2+^-ATPase or SERCA pump is involved in refilling ER Ca^2+^ storage. In addition, TG inhibits the SERCA-Ca^2+^-ATPase, which may affect the transport of Ca^2+^ in the ER (38, 39). In this study, the Δ[Ca^2+^]_cyt_ in the parental and *ΔTgpCaBP* parasite was not significantly affected by TG treatment (Fig. 5G); this may be because the parental parasite strain lost its regulatory effect on ER Ca^2+^ storage after TG was applied. The same phenomenon is observed in the *ΔTgpCaBP* parasite without TG treatment, suggesting that TgpCaBP may play a role in ER Ca^2+^ storage. Subsequently, we treated the parasites with Zap to induce a short-term increase in Ca^2+^ levels, Δ[Ca^2+^]_cyt_ in the *ΔTgpCaBP* parasites was significantly higher than that in the parental parasites (Fig. 5H), confirming the role of TgpCaBP in Ca^2+^ efflux.

**Fig 6:**
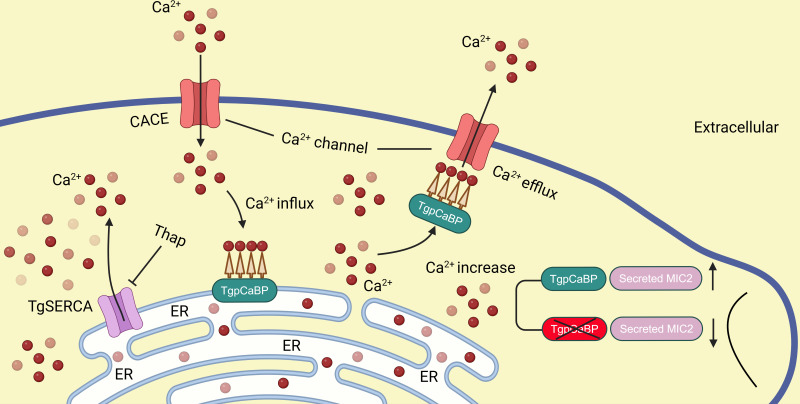
Schematic illustration of TgpCaBP in cytosolic Ca^2+^ efflux and regulatory function in ER Ca^2+^ storage in *T. gondii*. When intracellular calcium concentration is too high, TgpCaBP will participate in calcium efflux to maintain calcium balance. TgpCaBPs bind to free Ca^2+^ and transport them to the ER for storage. TgpCaBP participates in the regulation of the secretion of microneme proteins. The figure was designed by BioRender.

Previous studies have shown that the oscillations in intracellular calcium levels may regulate MIC2 secretion and parasite motility of *T. gondii* (32). Here, the cytosolic calcium level in the *ΔTgpCaBP* parasites was abnormally elevated (Fig. 5), and the invasion efficiency of the parasite was impaired. Deletion of TgpCaBP resulted in reduced cell invasion efficiency, slower egress from host cells, and decreased parasite motility (Fig. 3). These phenotypes are related to the imbalance in Ca^2+^ homeostasis caused by the *ΔTgpCaBP* parasite line, and the decreased efficiency of invasion and gliding motility may be directly caused by reduced secretion of MIC2, which was observed only in the *ΔTgpCaBP* parasites (Fig. 4). Animal infection experiments indicated that the virulence of the *ΔTgpCaBP* parasites was reduced (Fig. 3); however, this was not the only factor associated with parasite infectivity. Overall, our data revealed that TgpCaBP is a protein that essentially regulates calcium levels in *T. gondii* (Fig. 5 and 6).

### Conclusions

In this study, we identified a novel gene coding for a calcium-binding protein in *T. gondii*. We found that the *ΔTgpCaBP* parasites had defects in many biological aspects, such as host cell invasion, intracellular growth, egress from host cell, gliding motility, and microneme secretion. Thus, TgpCaBP is suggested to critically regulate the biological function of various proteins in the parasite apart from the modulation of several secretory proteins associated with parasite invasion and motility. Further studies are needed to detect the specific participation of TgpCaBP in the Ca^2+^ signaling pathway and better understand its functions and Ca^2+^ signaling.

## MATERIALS AND METHODS

### *Toxoplasma gondii* growth

The *T. gondii* RH strain was cultured in monkey kidney adherent epithelial (Vero) cells in Dulbecco’s modified minimal Eagle’s medium (DMEM; Servicebio), supplemented with 2% fetal bovine serum (FBS; Excell; Bio), and 100 µg/mL streptomycin (Beyotime) at 37 °C with 5% CO_2_.

### Bioinformatic analysis and molecular modeling

The gene sequence encoding TgpCaBP (gene ID: TGME49_229480) was downloaded from the *Toxoplasma gondii* database (ToxoDB). Structure predictions were performed using AlphaFold v2.3 (https://github.com/google-deepmind/alphafold/releases/tag/v2.3.0).

### Generation of specific antibodies

*T. gondii* cDNA fragments encoding pCaBP, decaprenyl diphosphate synthase (IPPS), and binding immunoglobulin protein (BIP) were amplified using polymerase chain reaction (PCR) with specific primers (Table S1). The amplification products were cloned into the pET-28a and pGEX-4T-1 vectors using the HB-infusion Cloning Kit (HANBIO) *via* EcoRI, BamHI, and NotI (TaKaRa). His and GST-tagged recombinant proteins were expressed and purified as previously described (40). The His-tagged recombinant proteins generated specific anti-TgpCaBP/TgIPPS/TgBIP polyclonal antibodies in rabbits and rats. Rabbits and rats were subcutaneously immunized four times with the recombinant protein emulsified in Freund’s adjuvant (Sigma); the initial immunization was performed using Freund’s complete adjuvant, and Freund’s incomplete adjuvant was used in subsequent immunizations. Accurately, 200 µg of recombinant protein was used for each immunization in rabbits, and 100 µg was used in rats. The final serum was harvested *via* cardiac puncture under ether anesthesia.

### Analysis of calcium-binding properties of native TgpCaBP

Harvested tachyzoites were resuspended in a cell lysis buffer (Beyotime) at 4 °C for 10 min (41) and centrifuged at 12,000 rpm for 10 min. To examine the electrophoretic mobility of TgpCaBP, 5 mM EGTA was added to the suspension to chelate free Ca^2+^, followed by the addition of 5 mM CaCl_2_. In the control group, MgCl_2_ or KCl was added instead of CaCl_2_. The supernatant was mixed with 5 × sodium dodecyl sulfate (SDS) loading buffer (Beyotime). The pellets were resuspended in phosphate-buffered saline (PBS) after three washes and mixed with loading buffer. The samples were loaded onto a 12% SDS gel. Rat anti-TgpCaBP polyclonal antibody (1:1,000) and the horseradish peroxidase-conjugated goat anti-rat immunoglobulin-G (1:2,000; EASYBIO) were used as the primary and secondary antibodies, respectively. Images were captured using a chemiluminescence imaging system (CLiNX).

### Indirect immunofluorescence assay of TgpCaBP in *T. gondii*

Extracellular parasites were collected and purified; *T. gondii* RH tachyzoites were washed twice with PBS, and 2 × 10^4^ tachyzoites were overlaid on adhesion microscope slides. Intracellular tachyzoites were grown in Vero cells on cell slides. Next, the tachyzoites were fixed with 4% paraformaldehyde for 20 min, permeabilized with 0.3% Triton X-100 for 10 min, blocked with 3% bovine serum albumin (BSA; Solarbio) for 1 h, and exposed to respective primary antibodies; subsequently, the nuclei were stained with 4′,6-diamidino-2-phenylindole (DAPI; Invitrogen) as previously described (42). The primary antibodies used were rat anti-TgpCaBP (1:500), rabbit anti-IPPS (1:500), and rabbit anti-BIP (1:500) (30); Alexa Fluor 488-conjugated goat anti-rat IgG (1:1,000; Thermo Fisher Scientific) and Alexa Fluor 594-conjugated goat anti-rabbit IgG (1:1,000; Thermo Fisher Scientific) were used as the secondary antibodies. For co-localization studies, rabbit anti-IPPS (1:500) and rabbit anti-BIP antibodies (1:500) were used as ER markers. Finally, images were captured using 100 × 1.5 oil immersion objectives on a SIM-ultimate microscope equipped with HIS-SIM IMAGER software.

### Generation of the *ΔTgpCaBP* and *ΔTgpCaBP-C* strains

To generate the *ΔTgpCaBP* parasites, we constructed the pSAG1-CAS9-U6-sgTgpCaBP plasmid using the Q5 Mutagenesis kit (New England Biolabs) and designed single-guide RNA (sgRNA) targeting *TgpCaBP* using a previously described method (43). The 5′ and 3′ untranslated regions (5′UTR and 3′UTR, repectively) of *TgpCaBP* were cloned to both ends of the dihydrofolate reductase (DHFR) cassette to construct the plasmid pTgpCaBP-DHFR. Accurately, 50 µg of the pSAG1-CAS9-U6-sgTgpCaBP plasmid and 10 µg of the DHFR cassette were co-transfected to *T. gondii* RH tachyzoites. Pyrimethamine (Sigma) was used for selection, and egressed tachyzoites were verified using PCR. The primers used are listed in Table S1.

To express the *TgpCaBP* gene in the *ΔTgpCaBP* parasites, we designed the plasmid p5’UPRT-TgpCaBP-3′UPRT. This plasmid was used to amplify a fragment containing the coding sequence of TgpCaBP, which is flanked by 5′UTR and 3′UTR of the uracil phosphoribosyl transferase (UPRT) gene. The pSAG1-CAS9-U6-sgUPRT plasmid was co-transfected with the amplified fragment. Fluorodeoxyribose (10 µM; Sigma) was used for selection. Parasites were tested by PCR, IFA, and western blotting.

### Quantitative PCR analysis of *TgpCaBP* in the parental, ME49, *ΔTgpCaBP,* and *ΔTgpCaBP-C* parasite strains

Total RNAs were extracted from parental, ME49, *ΔTgpCaBP,* and *ΔTgpCaBP-C* parasite strains and reverse transcribed into cDNA. The quantitative PCR (qPCR) was performed using monAmp chemoHS qPCR Mix (Monad) and quantitative real-time PCR was carried out on a QuantStudio 3 system (Thermo Fisher Scientific). The fold change was used to calculate the relative gene expression level of *TgpCaBP*, employing the 2^−ΔΔCT^ method (44). The primers that were used are listed in Table S2. The experiment included three biological replicates and three technical replicates. Actin and GAPDH were used as the housekeeping genes to reflect the level of transcription of each gene.

### Growth and invasion assays

Briefly, 200 egressed parasites were collected to infect Vero cells cultured in 6-well plates for 5–7 days. After three washes with PBS, cells were fixed with 4% paraformaldehyde for 20 min, and stained with crystal violet as previously described (45). Plaque sizes were analyzed using ImageJ software. For invasion assays, some changes were made to the previously described method (46). A monolayer of Vero cells was grown in a 12-well plate and infected with 2 × 10^6^ tachyzoites for 1 h. The cells were then washed and fixed with 4% paraformaldehyde. Attached parasites (uninvaded) were stained with rabbit anti-SAG1 polyclonal antibody (1:500) without permeabilization, and internal parasites (invaded) were stained with rat anti-TgpCaBP polyclonal antibody (1:500) after permeabilization. Subsequently, slides were stained with Alexa Fluor 488-conjugated goat anti-rabbit IgG and Alexa Fluor 594-conjugated goat anti-rat IgG (1:1,000; Thermo Fisher Scientific). Images were captured using a Leica DFC7000 T camera with LAS X software. The rate of invasion was determined by dividing the number of invaded parasites by the total number of parasites in the visual fields.

### Egress experiments

Vero monolayers were infected with 1 × 10^6^ tachyzoites in 12-well plates for 2 h and cultured at 37 °C with 5% CO_2_ for 24 h. Accurately, 3 µM Ca^2+^ ionophore A23187 (MCE) was added at 37 °C for 3 min and DMSO was used as a control. Subsequently, the monolayers were fixed with 4% paraformaldehyde for 20 min and blocked with 3% BSA for 30 min. Next, monolayers were exposed to rabbit anti-SAG1 (1:1,000) and Alexa Fluor 488-conjugated goat anti-rabbit IgG antibody (1:1,000; Thermo Fisher Scientific). The proportion of egressed versus intact vacuoles was determined using epifluorescence microscopy of 200 vacuoles per coverslip. The experiment consisted of three independent replicates.

### Replication experiments

Vero monolayers grown on glass coverslips were infected with 5 × 10^5^ freshly harvested parasites in 12-well plates for 2 h, washed twice to remove uninvaded tachyzoites, and developed in DMEM supplemented with 2% FBS at 37 °C with 5% CO_2_ for 20–24 h. Monolayers were covered with 4% paraformaldehyde and 0.25% Triton X-100 for 10 min each and then blocked with 3% BSA. The monolayers were stained with rabbit anti-SAG1 antibody (1:1,000) and secondary antibody Alexa Fluor 488-conjugated goat anti-rabbit IgG (1:1,000; Thermo Fisher Scientific). The number of parasites per vacuole was determined using a Leica DFC7000 T camera and the percentage of vacuoles with different numbers of parasites was calculated. All experiments were repeated thrice.

### Gliding experiments

Gliding experiments were performed as previously described (32). Freshly harvested parasites were resuspended in DMEM containing 3 µM A23187 (MCE) and glided on glass coverslips for 15 min in a 37 °C incubator. The parasites were fixed with 4% paraformaldehyde for 15 min, blocked with 5% skim milk powder for 1 h, incubated with rabbit anti-SAG2 antibody (1:500), and visualized by staining with Alexa Fluor 488-conjugated goat anti-rabbit IgG (1:1,000; Thermo Fisher Scientific). Three independent experiments were used to record the trails. The videos were shot with cellSens software for 10 min.

### Mice infection experiments with the parental, *ΔTgpCaBP,* and *ΔTgpCaBP-C* parasites

Eighty female BALB/c mice were divided into eight groups and intraperitoneally inoculated with 50, 500, 5 × 10^3^, or 5 × 10^5^ parental or *ΔTgpCaBP* parasites. After obtaining *ΔTgpCaBP* complemented strains, 50 *ΔTgpCaBP-C* tachyzoites were similarly inoculated and monitored for 28 days.

### Cytosolic Ca^2+^ measurements

The experiment was conducted according to a previously described technique (47, 48). Briefly, egressed tachyzoites were collected and washed thrice at 2,000 rpm for 10 min in Hank’s Balanced Salt Solution (HBSS, without Ca^2+^ and Mg**^2+^**, Beyotime). Parasites were suspended in HBSS buffer containing 2% saccharose to obtain a concentration of 1 × 10^9^ parasites/mL. The suspension containing 5 µM Fura-2 AM was placed in a water bath at 37 °C for 30 min and washed twice with HBSS buffer. The parasites were suspended in HBSS at a density of 1 × 10^9^ parasites/mL and kept on ice away from light. Fluorescence measurements were performed with a CYTATION 3 imaging reader (BioTeK), requiring 2 × 10^7^ parasites/mL per well. The excitation wavelengths were 340 and 380 nm and the emission wavelength was 510 nm. The measurements were subtracted from the background fluorescence values of the parasites at 340 and 380 nm and then calibrated according to the ratio of the fluorescence values of 340/380 nm, as previously described (49). Changes in Ca^2+^ concentration were calculated by subtracting the baseline from the measurements obtained for the first 20 s after the addition of Ca^2+^ or the first 100 s after the addition of drugs.

### Microneme secretion

First, 4 × 10^6^ tachyzoites were resuspended and fixed with various concentrations of A23187 (1 and 3 µM) or 1% DMSO for 5 min, and 2% ethanol was added ad the positive control (50). Secretion was stimulated at 37 °C for 5 min. Subsequently, the suspension was in an ice bath for 10 min to promote further secretion. Suspensions were centrifuged at 12,000 rpm for 15 min to collect the supernatants, and pellets were washed with PBS to remove the remaining secretions. Finally, the pellets were lysed by ultrasonication, and the samples were loaded onto 12% SDS gels.

### Statistics

All experimental data were obtained using at least three independent replicates. The data were analyzed in GraphPad Prism 9.0 using one- or two-way analysis of variance with Tukey’s multiple comparison test and Student’s *t*-test. Error bars represent the standard error of the mean.

## Data Availability

All data generated or analyzed are available in the manuscript and supplementary materials.

## SUPPLEMENTAL MATERIAL

Supplemental material spectrum.00661-24-s0001.docx Supplemental figures, tables, and video legends.

Video S1 spectrum.00661-24-s0002.mp4 Effect of A23187 on TgpCaBP knockout parasites.

Video S2 spectrum.00661-24-s0003.mp4 Effect of A23187 on WT T. gondii RH parasites.

Video S3 spectrum.00661-24-s0004.mp4 Effect of A23187 on TgpCaBP-C parasites.

ASM does not own the copyrights to Supplemental Material that may be linked to, or accessed through, an article. The authors have granted ASM a non-exclusive, world-wide license to publish the Supplemental Material files. Please contact the corresponding author directly for reuse.
