# Supplementary material for: Calcium-binding protein TgpCaBP regulates calcium storage of the zoonotic parasite Toxoplasma gondii
Source: Microbiol Spectr. 2024 Aug 20;12(10):e00661-24. doi: 10.1128/spectrum.00661-24 (PMC11448132; doi:10.1128/spectrum.00661-24)
Supplement: Supplemental material — Supplemental figures, tables, and video legends. [file spectrum.00661-24-s0001.docx]

**SUPLEMENTARY INFORMATION:**

**
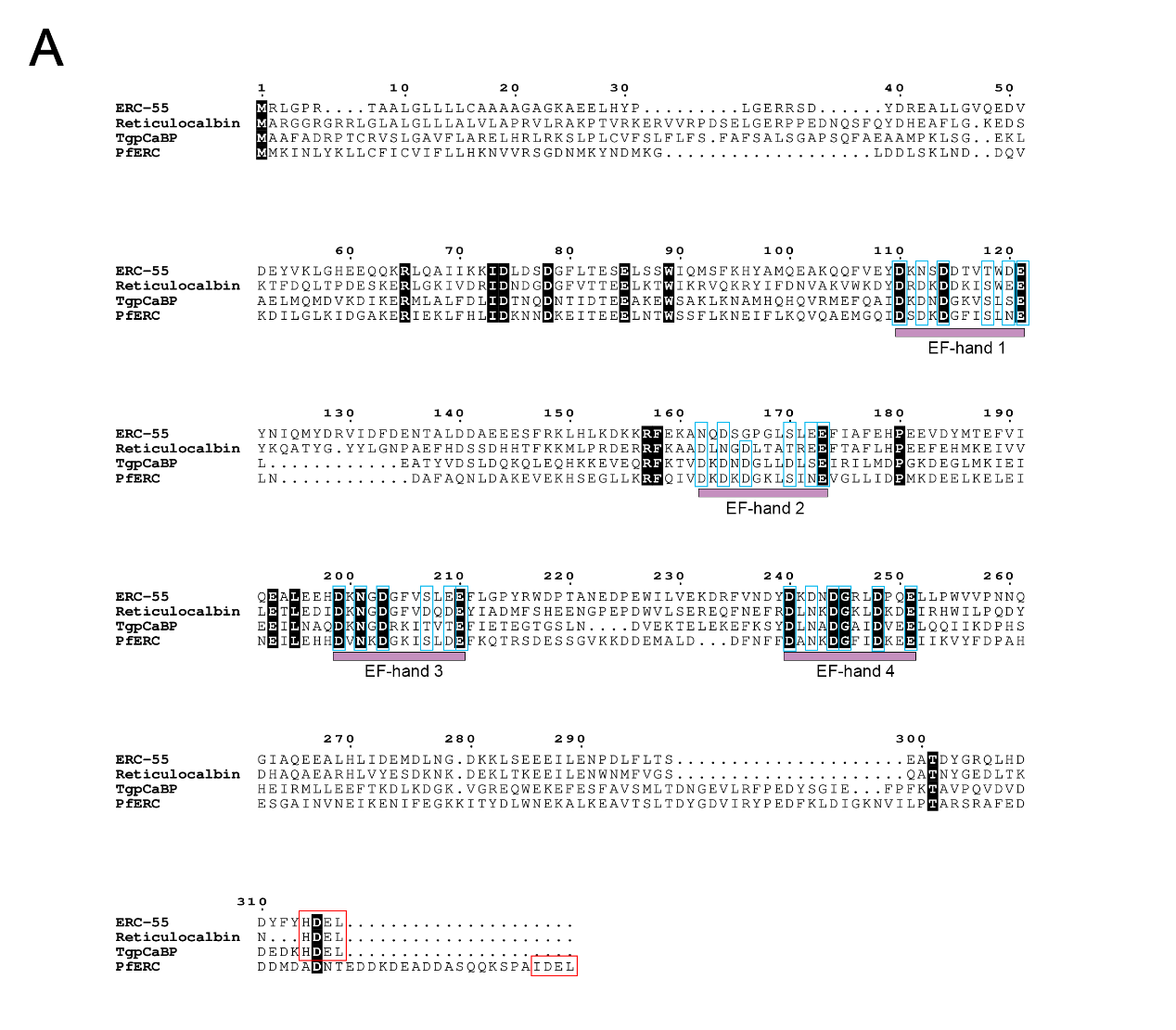
**

**Figure S1. Sequence alignment of TgpCaBP with other EF-hand calcium-binding proteins. (A)** Sequence alignment of human reticulocalbin, ERC-55, *Toxoplasma gondii* TgpCaBP and *Plasmodium falciparum* PfERC. The regions corresponding to the calcium-binding EF hand loops are indicated in purple color, the consensus sequences for the EF hand loops are boxed in blue and C-terminal HDEL motifs are boxed in red color.

**
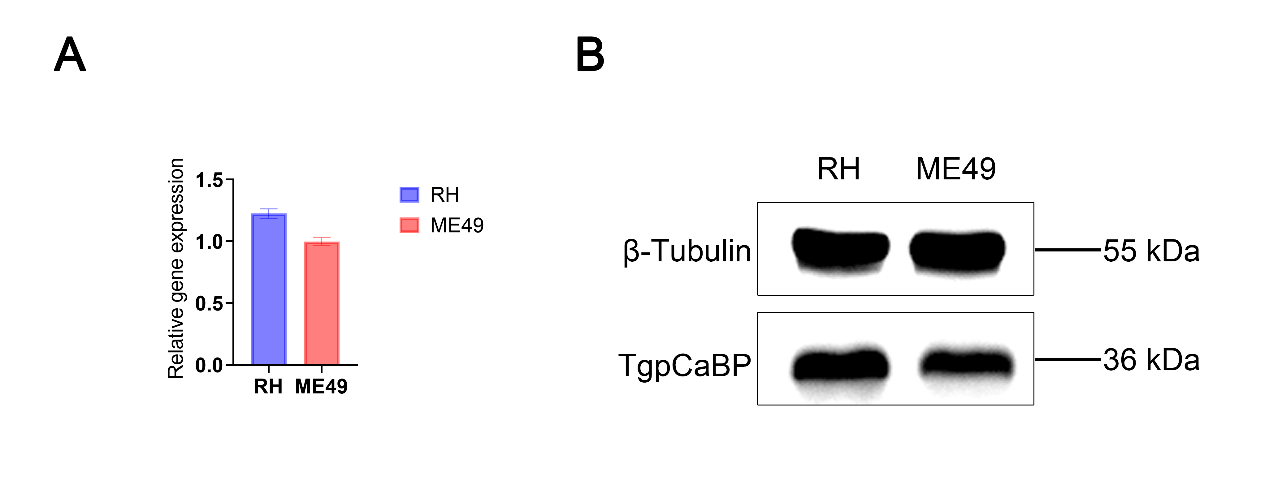
**

**Figure S2. Transcription and protein expression levels of TgpCaBP in RH and ME49 strains.** (A) The quantitative PCR analysis of TgpCaBP transcription level in RH and ME49 strains. The result showed that the transcription level of TgpCaBP in RH strain was higher than that in ME49 strain. (B) The relative protein expression level of TgpCaBP in RH and ME49 strains. The expression level of TgpCaBP in RH strain was higher than that in ME49 strain, and β-tubulin was used as a reference control.


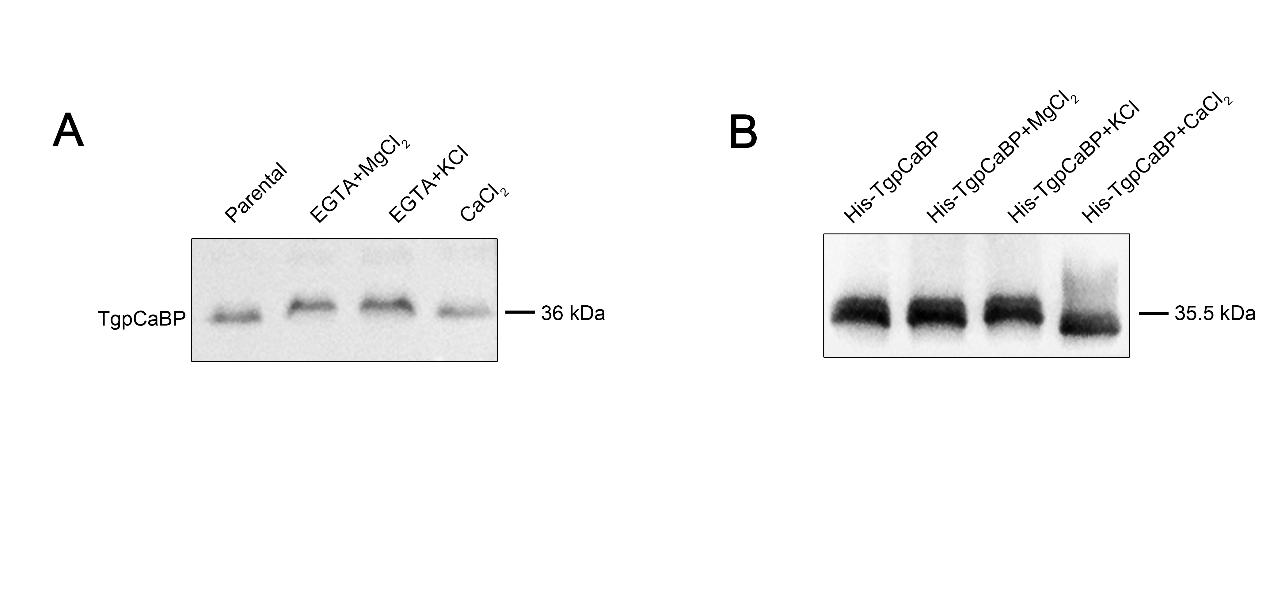


**Figure S3. Mg^2+^ and K^+^ do not affect the electrophoretic mobility of TgpCaBP.** (**A** and **B**) Only Ca^2+^ affects the electrophoretic mobility of both the native TgpCaBP and the recombinant His-TgpCaBP, while Mg^2+^ and K^+^ do not have this effect.

**
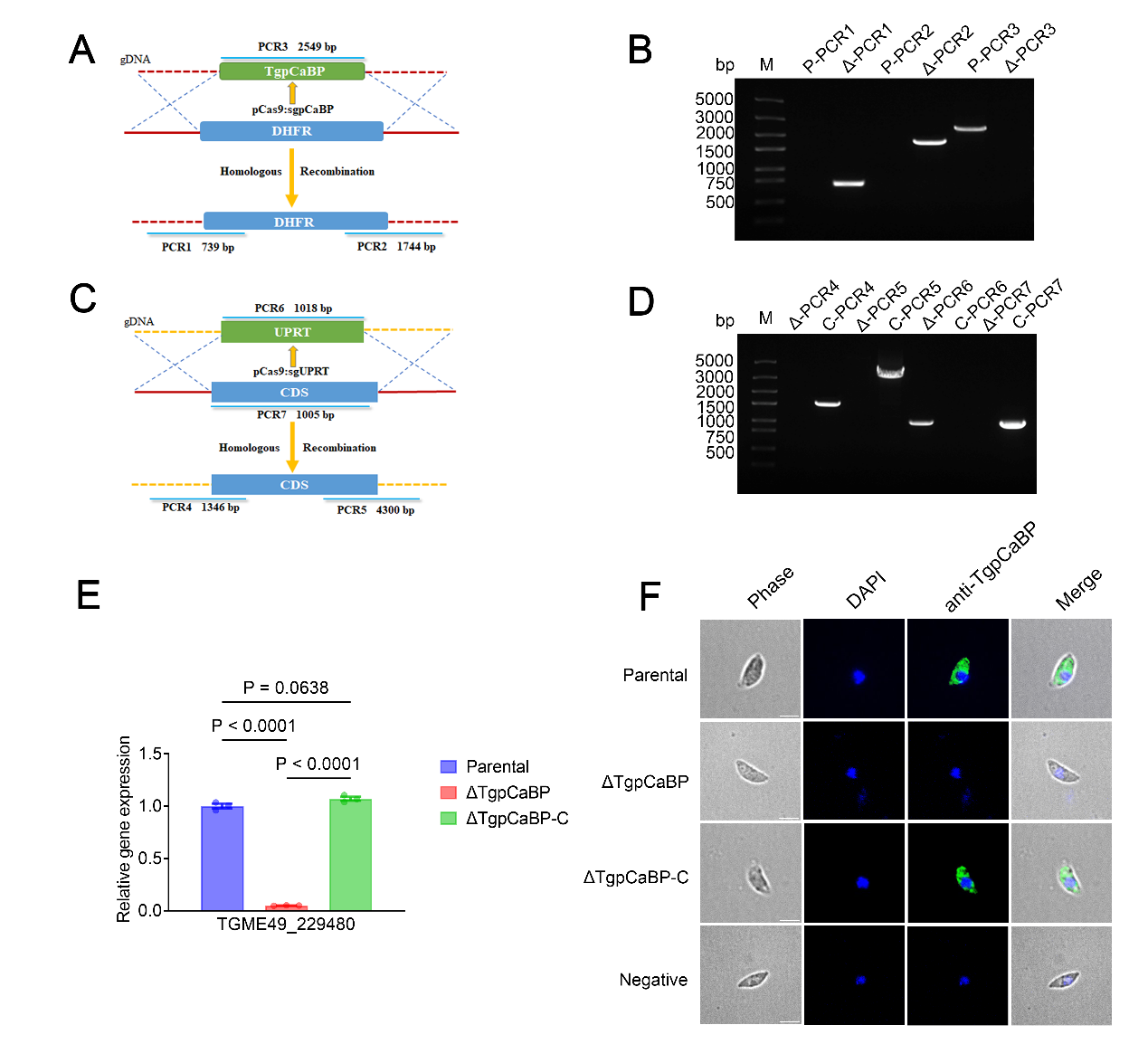
 Figure S4.** **The CRISPR/Cas9 strategy and verification of ΔTgpCaBP and ΔTgpCaBP-C.** (**A**) The strategy of generation of *TgCaBP* kockout parasite, ΔTgpCaBP. (**B**) Verification of ΔTgpCaBP by PCR. The PCR of ΔTgpCaBP (Δ-PCR1+, Δ-PCR2+, Δ-PCR3-, positive control) and the PCR of RH (P-PCR1-, P-PCR2-, P-PCR3+, negative control) indicate that the *TgpCaBP* gene was replaced by DHFR cassette. (**C**) The strategy of generation of *TgCaBP* complement strain, ΔTgpCaBP-C. (**D**) *TgpCaBP* gene was reintroduced into the genome of the ΔTgpCaBP mutant. PCR of ΔTgpCaBP-C (C-PCR4+, C-PCR5+, C-PCR7+, and C-PCR6-) demonstrated that the *TgpCaBP* gene was complemented at the UPRT locus of the ΔTgpCaBP mutant. (**E**) The qPCR of *TGME49_229480* showed a significantly reduced transcription level in the ΔTgpCaBP parasites and the transcription levels in the parental and the ΔTgpCaBP-C parasites were shown to be normal. (**F**) Immunofluorescence analysis of the expression of TgpCaBP protein in the extracellular parental, ΔTgpCaBP and ΔTgpCaBP-C parasites. The ΔTgpCaBP parasites lost the expression of TgpCaBP compared to the other two parasite lines. Scale bar, 5 μm. Abbreviation: DAPI, 4’,6-diamidino-2-phenylindole.

**Legends for the supplemental videos**:

**Video 1**: **Effect of A23187 on *TgpCaBP* knockout parasites**. The *TgpCaBP* knockout parasites showed decreased sensitivity to the treatment of calcium ionophore A23187 in mobility.

**Video 2**: **Effect of A23187 on wild type (WT) *T. gondii* RH parasites**. The mobility of WT *T. gondii* parasites was enhanced by the calcium ionophore A23187.

**Video 3**: **Effect of A23187 on *TgpCaBP-C* parasites**. The mobility of *TgpCaBP-C* *T. gondii* parasites was enhanced by the calcium ionophore A23187.

**Table S1: Primers used for gene cloning, generation of knockout and gene complementary parasites:**

| Primers | Sequence (5'-3') | Used for |
| --- | --- | --- |
| TgIPPS _primer F | aatgggtcgcggatccGACGTGACGATACCTCGG | To amplify the coding sequence of TgIPPS |
| TgIPPS _primer R | agtgcggccgcaagcttGAAGAACCGGAAGCATCT |  |
| TgBIP _primer F | caaatgggtcgcggatccAGCGTGGCTACTCTCCGA | To amplify the coding sequence of TgBIP |
| TgBIP _primer R | agtgcggccgcaagcttCTGCAAACCGCGTTTGTC |  |
| TgpCaBP _primer F | TGGGTCGCGGATCCGAATTCATGCAGATGGACGTGAATTT | To amplify the coding sequence of TgpCaBP |
| TgpCaBP _primer R | agtgcggccgcaagcttCAGTTCATCGTGTTTGTCTTT |  |
| sgRNA-TgpCaBP | GCGATCGACAAAGACAACGA  GTTTTAGAGCTAGAAATAGC | To mutagenize the sgRNA |
| sgRNA-R | AACTTGACATCCCCATTTAC | To mutagenize the sgRNA |
| RH, KO-PCR1 F | AGGCGGGTGAGCGTGGGT | To examine the integration at 5'-UTR of TgpCaBP locus, 739 bp |
| RH, KO-PCR1 R | GTTAGTCCCCTGGCACGAGAGATAG |  |
| RH, KO-PCR2 F | TCTTTTCGGAGGGATCAGGGA | To examine the integration at 3'-UTR of TgpCaBP locus, 1744bp |
| RH, KO-PCR2 R | GCAATGCCAAGGTGGTGAATG |  |
| RH, KO-PCR3 F | GTCTCTGGGTGCGGTCTTCCT | To confirm the replacement of *TgpCaBP* by *DHFR.* |
| RH, KO-PCR3 R | CAGTTCATCGTGTTTGTCTTCGTC |  |
| KO, C-PCR4 F | TGTCCCTGGGTGTTTCCT | To test the integration at 5'-UTR of UPRT locus 1,346 bp |
| KO, C-PCR4 R | CTGTTCTGCGATGAGC |  |
| KO, C-PCR5 F | CCGTTGTGCTCACTTCTC | To test the integration at 3'-UTR of UPRT locus 4,300 bp |
| KO, C-PCR5 R | GCCTTGGTCATGTGCCTT |  |
| KO, C-PCR6 F | CATCATCACGAGGTAATCC | To confirm the replacement of *UPRT* by *TgpCaBP,*  1,018 bp |
| KO, C-PCR6 R | GCAGTCGCACAGAAACAT |  |
| KO, C-PCR7 F | GTCTCTGGGTGCGGTCTTCCT | To confirm the insertion of *TgpCaBP* at the *UPRT* locus, 1,005 bp |
| KO, C-PCR7 R | CAGTTCATCGTGTTTGTCTTCGTC |  |

**Table S2: Primers used for qPCR:**

| Primers | Sequence (5'-3') |
| --- | --- |
| TgpCaBP _primer F | AGCTACGATTTGAATGCCGACG |
| TgpCaBP _primer R | TCCTTCTCCCACTGCTCCCT |
| Actin _primer F | TGCCGTCTTCCCGTCTATCG |
| Actin _primer R | TTTTCTCCATGTCGTCCCAGTT |
| GAPDH _primer F | ATTTTGCTTGGGATTCGAGGA |
| GAPDH _primer R | TGCAGGGTAACGATCAAAAAATG |
